# Inequalities in the incidence of cervical cancer in South East England 2001–2005: an investigation of population risk factors

**DOI:** 10.1186/1471-2458-9-62

**Published:** 2009-02-20

**Authors:** Laura G Currin, Ruth H Jack, Karen M Linklater, Vivian Mak, Henrik Møller, Elizabeth A Davies

**Affiliations:** 1 King's College London, Thames Cancer Registry, 1st Floor, Capital House, Weston Street, London, SE1 3QD, UK

## Abstract

**Background:**

The incidence of cervical cancer varies dramatically, both globally and within individual countries. The age-standardised incidence of cervical cancer was compared across primary care trusts (PCTs) in South East England, taking into account the prevalence of known behavioural risk factors, screening coverage and the deprivation of the area.

**Methods:**

Data on 2,231 cases diagnosed between 2001 and 2005 were extracted from the Thames Cancer Registry, and data on risk factors and screening coverage were collated from publicly available sources. Age-standardised incidence rates were calculated for each PCT using cases of squamous cell carcinoma in the screening age group (25–64 years).

**Results:**

The age-standardised incidence rate for cervical cancer in South East England was 6.7 per 100,000 population (European standard) but varied 3.1 fold between individual PCTs. Correlations between the age-standardised incidence rate and smoking prevalence, teenage conception rates, and deprivation were highly significant at the PCT level (*p *< 0.001). However, screening coverage was not associated with the incidence of cervical cancer at the PCT level. Poisson regression indicated that these variables were all highly correlated and could not determine the level of independent contribution at a population level.

**Conclusion:**

There is excess disease burden within South East England. Significant public health gains can be made by reducing exposure to known risk factors at a population level.

## Background

Cervical cancer represents a significant public health concern. Worldwide, cervical cancer is the second most common malignancy among females [1]. Although the 5-year survival rates for this cancer are relatively high, on average women are diagnosed and die at a younger age than in most other types of cancer. The total years of life lost due to this illness are therefore substantial. Currently, cervical cancer rates eighth in terms of cancer incidence in the United Kingdom [2].

Within the United Kingdom, rates of cervical cancer are highest in the Yorkshire and North West regions of England, and Scotland; and lowest in the Eastern, South East and London regions of England [2]. Within London, a recent report highlighted the link between socio-economic deprivation and inequalities in cervical cancer incidence and mortality [3]. Age-standardised incidence rates per 100,000 European population were 9.6 in the most deprived areas compared to 5.4 in the most affluent areas. Age-standardised mortality rates were 4.2 and 1.7 per 100,000 for the most deprived and most affluent areas respectively. This relationship between deprivation and higher incidence of cervical cancer is consistently replicated [4,5] even when controlling for access to medical care [6].

The Cancer Reform Strategy published by the Department of Health has recently prioritised the reduction of cancer inequalities across England [7]. To target public health interventions it is important to know why deprivation is associated with increased rates of cervical cancer. For example, is deprivation itself a predictor of cervical cancer, or can the association be explained by a relationship between deprivation and known aetiological risk factors?

In the aetiology of cervical cancer, the human papilloma virus (HPV) is a necessary precursor for carcinogenesis and is found in almost all cases of squamous cell carcinomas [8]. However, infection alone is insufficient to result in cervical cancer as infection rates are approximately 1,000 times higher than the incidence of cervical cancer and at least 90% of infections resolve spontaneously [9,10]. Established co-factors for the development of cervical cancer include multi-parity, age at first delivery, tobacco use, and use of oral contraceptives [11-14].

Both HPV infection rates and the prevalence of co-factors within a population are known to be influenced by socio-economic variables. For example, in a recent study conducted in the United States women living below the poverty line had almost twice the rate of HPV compared to those living above (23% versus 12%, *p *= .03) [15]. Part of this relationship may be explained by different sexual behaviour. Sexual contact determines exposure to HPV, and early sexual intercourse has been identified as a risk factor for the development of cervical cancer [16].

Epidemiological studies implicate cigarette smoking as an independent risk factor for the development of cervical cancer, with odds ratios ranging from 1.8 to 4.3 [17,18]. This effect appears to be limited to the squamous cell subtype of cervical cancer [17], although the exact biological mechanism is still debated. Smoking is likely to lead to cervical cancer through multiple pathways. There is evidence of alteration of the tumour suppressor gene *FHIT *[19], inhibition of normal cervical cell proliferation [20], and local immune suppression resulting in persistent HPV infection [21]. Smoking rates in a population are known to be strongly predicted by socio-economic deprivation [22]. Therefore, it is likely that the association between cervical cancer and deprivation could be partially explained by differential rates of smoking.

Socio-demographic features of a population may also affect prevention efforts. Screening programmes are effective in reducing the incidence and mortality of cervical cancer by detecting early cellular changes which could lead to cervical cancer [23]. There is a 63% predicted reduction in lifetime incidence in populations with an effective screening program compared to unscreened populations [24]. In England, the National Health Service Cervical Screening Programme (NHSCSP) was established in 1988 and the current target for screening coverage is 80% of all eligible females in the general practice population (screened at variable, age-dependent, intervals) [25,26]. However, screening utilisation is influenced by socio-demographic variables. For example, women from disadvantaged backgrounds are more likely to see the screening procedure as aversive and feel less obliged to attend than their more well off neighbours [27]. In the UK, cervical screening coverage is consistently higher in affluent areas, although this disparity is declining [28,29].

This study aimed to quantify the relationship between cervical cancer incidence and aetiological risk factors and screening coverage at a population level; and to investigate whether deprivation itself predicts an additional risk to the population studied. The specific objectives were:

1. To demonstrate geographical inequalities in the age-standardised incidence of cervical cancer in the area of South East England covered by the Thames Cancer Registry (TCR) between 2001 and 2005.

2. To use Spearman's correlation coefficient to investigate the association at a population level between incidence and aetiological risk factors (i.e. sexual behaviour, smoking); and incidence and screening coverage.

3. To use Poisson regression to determine if deprivation remains an independent risk factor for the development of cervical cancer when controlling for these variables.

## Methods

In the United Kingdom cancer registries record the occurrence of cancer in their resident populations. During the study period the Thames Cancer Registry (TCR) covered a population of 12 million people living in the area including London, Kent, Surrey and Sussex. In this area, cancer registration is initiated by clinical and pathological information received from hospitals and by information about deaths provided by the National Health Service Central Register through the Office for National Statistics. Trained data collection officers collect further demographic information, details on disease stage and treatment received in the six months following diagnosis from the medical records of individual cases. Data are continuously added to a central database and quality assured. To prevent double counting, information about new tumours is cross-checked against already registered cases.

Information on tumour stage is extracted from the medical records, however, since this information is often not complete, TCR creates an in-house classification using all available information to approximate stage at diagnosis. Tumours are categorised as 'local' (stage 1), 'extension beyond organ of origin' (stage 2), 'regional lymph node involvement' (stage 3) and 'metastasis' (stage 4). Tumours with insufficient information are classified as having disease stage 'not known'.

The first objective of the study was, to describe inequalities in South East England. Data were extracted from the TCR database on 2,231 cases diagnosed with cervical cancer [ICD-10 category C53; [30]] between 2001 and 2005. Descriptive statistics defined the sample by tumour type and the stage of presentation. Cases were stratified into five-year age bands to calculate age-specific incidence rates for cervical cancer for the years 2001–2005. An age-standardised incidence rate was calculated for women living within the entire catchment area using the European standard population.

The geographic area covered by the TCR is diverse, including urban, suburban and rural areas and areas of contrasting affluence and deprivation. Thirty-one of the constituent primary care trusts (PCTs) are within the London Strategic Health Authority and eight within the South East Coast Strategic Health Authority. To investigate inequalities in the incidence of cervical cancer, the age-standardised incidence was calculated for each PCT. The age-standardised incidence was calculated for women of all ages, and then restricted to cases of squamous cell carcinomas within women aged 25–64 years to include only the screened age group. Squamous cell cases were considered specifically because this histological type is associated with the risk conferred by smoking and also the most likely to be detected by screening [17]. Calculation of truncated age-standardised rates is described by Boyle and Parkin [31]. Truncated incidence rates were used for all between PCT comparisons, and a chi square test of heterogeneity was used to establish the statistical significance of the variation between PCTs.

The second objective of this study was to assess the relationship between age-standardised incidence and aetiological risk factors or prevention efforts at the population level. Although HPV infection rates are not routinely measured, teenage conception rates can act as a proxy for the early sexual contact which confers a behaviour risk for cervical cancer [32]. One potential limitation of this approach is the potential for teenage conception to be confounded with other HPV co-factors such as tobacco use or age at first delivery. However, teenage conception rates are included as a health indicator by the Health Care Commission [33] and therefore reliably measured and reported at the PCT level. Teenage conception rates for the PCTs covered by the TCR were obtained from the Office for National Statistics (ONS) and then correlated with the truncated age-standardised incidence rates by PCT. Spearman's correlation coefficient was used to test whether these two factors were correlated.

National and regional figures for smoking prevalence are regularly produced, yet figures for individual PCTs rely on synthetic statistical estimation [34]. These synthetic estimates were used to correlate PCT smoking prevalence with the age-standardised incidence, using Spearman's correlation coefficient.

Cervical screening coverage within PCTs was obtained from the ONS [35]. Screening coverage was calculated by considering the number of women screened divided by the total eligible population within the target age range (25 to 64) during the 2004/2005 financial year. ONS figures were used in preference to those reported by the Quality and Outcomes Framework, as the latter are based on remuneration figures for primary health care providers and allow for some reductions in target population figures. Spearman's correlation coefficient was calculated to assess the relationship between the age-standardised incidence and screening coverage at the PCT level.

Cases were assigned into deprivation categories using super-output areas, which are based on the postcode of residence. The income domain of the Indices of Deprivation 2004 was used to classify super-output areas into deprivation quintiles in which 1 is the most affluent and 5 is the most deprived [36]. PCTs were ranked according to the percentage of areas within their borders in the two most deprived quintiles.

Finally, Poisson regression was used to model the incidence of cervical cancer at the PCT level. The aim of this analysis was to determine if deprivation predicted a risk over and above that contributed by teenage conception, smoking, and screening. All analyses were adjusted for age and PCT.

## Results

### Tumour description

Table 1 illustrates the histological classification of incident cases of cervical cancer in the TCR in the years between 2001 and 2005. Squamous cell carcinoma was the most common presentation (63.9%; n = 1,425). Missing data prevented determination of tumour stage in 34.5% (n = 770) of cases. However of the cases with sufficient information, the majority (69.3%; n = 1,012) presented with localised disease (data not presented).

**Table 1:** Histological classification of cervical cancer diagnosed in women in South East England, 2001–2005

**Classification**	**Count (%)**
Squamous cell carcinoma	1,425 (63.9)
Adenocarcinoma	497 (22.3)
Unknown	251 (11.3)
Other specified histologies	58 (2.6)
**Total**	**2,231 (100)**

### Age-specific incidence

**Figure 1:**
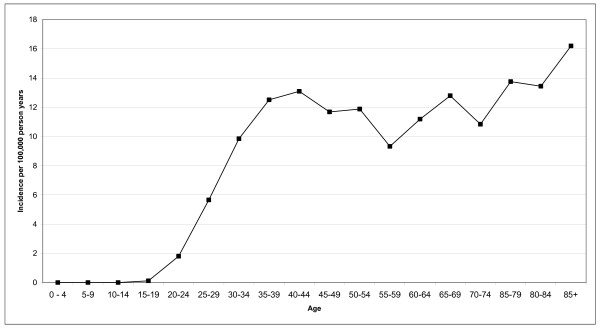
**Age-specific incidence rates of cervical cancer in women in South East England, 2001–2005**.

Analysis of the age-specific incidence showed a marked rise in cervical cancer incidence in the third and fourth decade of life, followed by a relative plateau (Figure 1).

### Age-standardised incidence

The age-standardised incidence rate for cervical cancer in women living in South East England was 6.7 per 100,000 person years using the European standard population for the years 2001–2005.

A test of heterogeneity demonstrated significant PCT variability in the truncated age-standardised incidence of squamous cell cervical cancer in the potential screened population (women age 25–64; χ^2 ^(40) = 103.4; p < 0.0001). The highest incidence (11.6 per 100,000 person years) was found in Lambeth PCT, and the eight PCTs with the highest incidence were all found within London rather than in suburban or rural environments. Surrey PCT had the lowest incidence (3.7 per 100,000 person years).

### Deprivation

**Figure 2:**
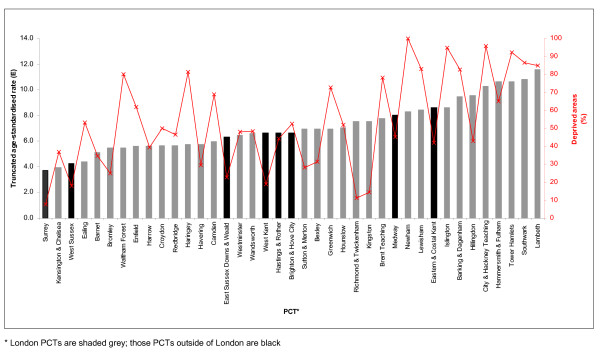
**Deprivation (red line) and age-standardised incidence of squamous cell cervical cancer (bars) in women aged 25–64 by PCT in South East England, 2001–2005**.

There was considerable variability in the underlying socio-economic status of the PCT populations. For example, in Surrey PCT only 8% of the super-output areas were in the two most deprived quintiles, compared with Newham PCT where all super-output areas were deprived. Figure 2 demonstrates that those PCTs with a higher proportion of deprived areas also had a higher incidence of cervical cancer. Deprivation was highly correlated with the truncated incidence of cervical cancer (Spearman r = 0.57; *p *< 0.001).

### Risk and prevention

In addition to differences in socio-economic status, PCTs also varied in terms of known risk factors (teenage conception rates, smoking prevalence) and prevalence of preventative measures (cervical screening coverage).

**Figure 3:**
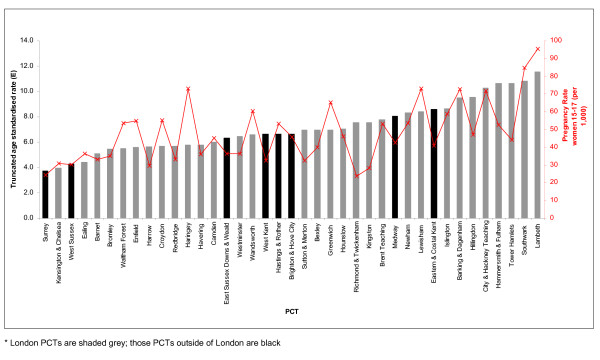
**Teenage conception (red line) and age-standardised incidence of squamous cell cervical cancer (bars) in women aged 25–64 by PCT in South East England, 2001–2005**.

The conception rates for women aged 15–17 varied over four-fold. Richmond & Twickenham PCT had the lowest rates of 23.6 per 1,000 population, compared to 95.2 per 1,000 in Lambeth PCT (Figure 3). There was a significant correlation between teenage conception rates and the age-adjusted incidence of squamous cell cervical cancer (Spearman r = 0.65; *p *< 0.001).

**Figure 4:**
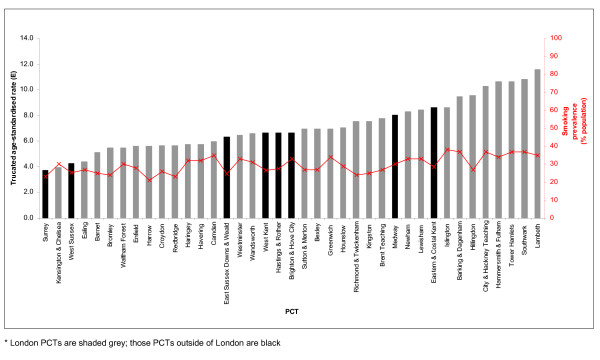
**Smoking prevalence (red line) and age-standardised incidence of squamous cell cervical cancer (bars) in women aged 25–64 by PCT in South East England, 2001–2005**.

Smoking prevalence in the TCR area varies from a low of 21% in Harrow PCT to a high of 38% in Islington PCT (Figure 4). There was a significant positive correlation between the age-standardised incidence of cervical cancer and smoking prevalence at PCT level (Spearman *r *= 0.62; *p *< 0.001).

**Figure 5:**
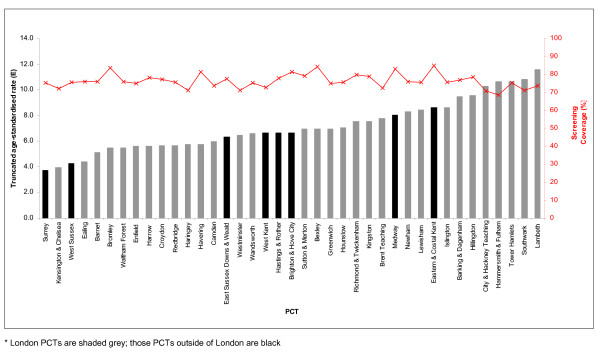
**Screening coverage (red line) and age-standardised incidence of squamous cell cervical cancer (bars) in women aged 25–64 by PCT in South East England, 2001–2005**.

PCT screening programmes had different levels of coverage. Screening of eligible women was lowest in Hammersmith & Fulham PCT (68.7%) and highest in Bexley PCT (84.3%). Figure 5 illustrates the non-significant negative correlation between age-standardised incidence and PCT screening coverage (r = -0.09; *p *= 0.61).

### Model of population risk

Table 2 presents the results from the Poisson regression analyses. In the univariate analysis each individual population variable significantly predicted the PCT incidence of cervical cancer (adjusted for age). However, when the four variables were considered together in the multivariate analysis, the only predictor which remained independently significant was smoking prevalence (*p *< 0.05).

**Table 2:** Predictors of the incidence of squamous cell cervical cancer at PCT level in South East England, 2001–2005

Univariate analysis*	**IRR**	**z**	***p***	**95% CI**
Deprivation	1.01	3.5	**<0.001**	1.00 – 1.01
Teenage conception	1.01	4.8	**<0.001**	1.01 – 1.02
Smoking prevalence	1.04	4.5	**<0.001**	1.02 – 1.06
Screening coverage	0.97	-2.6	**0.008**	0.95 – 0.99

**Multivariate analysis**^**†**^	**IRR**	**z**	***P***	**95% CI**

Deprivation	1.00	0.28	0.78	1.00 – 1.00
Teenage conception	1.01	1.76	0.08	1.00 – 1.01
Smoking prevalence	1.02	2.06	**0.04**	1.00 – 1.02
Screening coverage	1.00	-0.42	0.67	0.97 – 1.00

* adjusted for age and PCT

^† ^adjusted for age, PCT, deprivation, smoking, teenage conception, and screening coverage

## Discussion

### Main findings

The age-standardised incidence rate for cervical cancer in South East England is 6.7 per 100,000 European standard population. When restricting the analysis to squamous cell carcinoma, which is the histological type most likely to be influenced by smoking prevalence and screening, there were significant inequalities in incidence within the area. There was a 3.1 fold difference between primary care trusts (PCTs) with the highest and lowest incidence. Age-standardised incidence rates of cervical cancer were significantly correlated with deprivation, teenage conception rates, and smoking prevalence. Attempts to model the independent contribution of these risk factors at a population level suggested that all these variables are highly correlated with one another, and this potential confounding limits the conclusions about the contribution of specific risk factors at the population level.

When looking at population studies of cervical cancer, deprived areas are known to have an increased incidence of cervical cancer [3-5,15]. This relationship remains even when controlling for availability and access to medical services [5], suggesting that these populations have differential exposure to risk factors important in the development and progression of the disease. These risk factors are potentially modifiable at the population level using public health interventions. For example, differences in sexual behaviour can influence the development of cervical cancer due to the transmission of HPV, and this variable sexual behaviour has been used to explain the vast inequalities between countries [37] and within populations [38]. Randomised trials have shown that population-based interventions can reduce risky sexual behaviour [39]; however the subsequent reduction in incidence of sexually transmitted infections has not been established [40].

Although smoking prevalence has been declining, areas of lower socio-economic status have a disproportionate number of active smokers. Smoking is known to be an independent risk factor for cervical cancer [18], with the exact biological mechanism yet to be determined. Smoking cessation has been the target of a major public health campaign in the UK, and the National Institute for Clinical Health and Excellence has produced recommendations for effective interventions [41]. By targeting these interventions in deprived areas with high smoking incidence one benefit could be the reduction of inequalities in the incidence of cervical cancer.

In this study, the lack of correlation between population screening and incidence of cervical cancer could be due to relatively uniform levels of screening in all PCTs studied, with the lowest levels above 68% of eligible females. While this homogeneity may mask a significant association between screening and incidence, it does highlight that a number of PCTs are below the Department of Health target of 80% screening coverage. Improvements in screening coverage at the population level could result in further reductions in the incidence and mortality of this disease [23]. Creative approaches may be needed to increase participation in those groups who will benefit most from screening, including lower socio-economic groups and those who smoke. Arguments have also been made for changes in the delivery of screening programmes to improve the yield of positive results and reduce the rate of false positives [7,23]. The emphasis on screening should continue despite advances in HPV vaccination programmes. HPV vaccination of girls aged 12 to 13 years began in the UK in September 2008 [42]. However, the time lag between infection and carcinogenesis means the vaccination programme will not result in a decline in cervical cancer incidence at a population level for at least a decade, underlining the importance of continued screening.

### Limitations

This study makes use of one of the largest population-based cancer registries in Europe to investigate inequalities in cancer incidence in a geographically defined area. Publicly available data were used to investigate known risk factors, including smoking and social deprivation. However, reliance on existing datasets limits the flexibility and range of analysis to variables that are currently available at the PCT level.

When attempting to explain inequalities in cervical cancer incidence it is important to consider all the known aetiological risk factors. The most important risk factor in the development of cervical cancer is infection with human papilloma virus (HPV) [8,37,43]. However, when analysing cervical cancer rates at a population level there are several difficulties quantifying concurrent or previous HPV infection. First, HPV infection is widespread and infection rates range from 4–40% in sexually active women with normal cervical cytology, yet most HPV infections resolve without leading to detectable lesions [9,44,45]. Second, HPV infection rates are not routinely collected from representative populations. Although infection with another sexually transmitted infection such as genital warts could be a useful proxy measure, this data is not available at the PCT level. This is an avenue for further research.

Synthetic estimates of smoking prevalence are not as robust as actual surveillance of the behaviour of the population of interest. Measures were in place to ensure consistency and reliability in these estimates [34], but the methodology might introduce a degree of confounding when considered in the context of other population characteristics. Synthetic estimates are calculated from the demographic and social characteristics of the area and they may therefore prove unreliable for inclusion in models of risk which also include these variables [46].

Screening participation rates at the population level are unlikely to represent adequately the variability in screening behaviours which may influence the development of cervical cancer. Optimal screening periods are variable by age [25,47], and this study does not take into account this factor in analysis, nor does it consider that many of the women screened may still be at an increased risk due to longer periods between screening.

## Conclusion

There are inequalities in the incidence of cervical cancer in South East England, and higher rates are clearly associated with deprivation. Public health campaigns could decrease the disease burden of cervical cancer by focusing on known aetiological risk factors. However, these risks cluster together and it is difficult to determine the individual contribution of each variable at a population level. The proximity of areas of high and low incidence demonstrate that population risk factors for cervical cancer can vary dramatically within a region. In the future other data, such as that on sexually transmitted infections, needs to be reported at a level of detail which is not currently available. Using better information on which individuals within a population have increased risk of cervical cancer, screening programmes and preventative efforts could be targeted more effectively.

## Competing interests

The authors declare that they have no competing interests.

## Authors' contributions

LGC conceived the project, analysed data analysis and drafted the manuscript; RHJ, KML, and VM assisted with data analysis and interpretation; HM and EAD assisted with data interpretation and critically revised the manuscript. All authors contributed to the study design and approved the final manuscript.

## Pre-publication history

The pre-publication history for this paper can be accessed here:


